# Editorial: AI and data science in drug development and public health: Highlights from the MCBIOS 2022 conference

**DOI:** 10.3389/fdata.2023.1156811

**Published:** 2023-02-27

**Authors:** Ramin Homayouni, Prashanti Manda, Aik Choon Tan, Zhaohui S. Qin

**Affiliations:** ^1^Department of Foundational Medical Studies, Oakland University William Beaumont School of Medicine, Rochester, MI, United States; ^2^Department of Informatics and Analytics, University of North Carolina at Greensboro, Greensboro, NC, United States; ^3^Department of Oncological Sciences and Biomedical Informatics, Huntsman Cancer Institute, Salt Lake City, UT, United States; ^4^Department of Biostatistics and Bioinformatics, Rollins School of Public Health, Emory University, Atlanta, GA, United States

**Keywords:** deep learning, NLP, genomics (G), regulatory science research, metagenomics

This Research Topic is a product of the 18th annual conference of the MidSouth Computational Biology and Bioinformatics Society (MCBIOS), which has a broad membership of scientists and trainees with research interests in genomics, medicine, and regulatory sciences. The topic includes a total of nine papers appearing in *Frontiers in Artificial Intelligence* (Medicine and Public Health), which include five original research articles, two methods articles, one brief research report and one review article. The papers can be categorized into four general themes of regulatory sciences, genomics, protein modeling and natural language processing, as detailed below.

## Regulatory science

The field of Artificial Intelligence (AI) has advanced significantly during the past few years, but its application to biomedical research, healthcare and regulatory sciences is still emerging. In particular, application of AI tools in regulatory decision-making and for drug safety and efficacy is not widely accepted, in part due to the perception that larger amount of data are needed to train accurate AI models. In their review article, Connor et al. challenge this perception with respect to adaptability of AI models on unseen data, focusing on evaluation of DeepDILI for predicting drug-induced liver injury (DILI). They concluded that the target test set plays a major role in assessing the adaptive behavior of AI models, but the training set does not significantly affect the predictive performance of the adaptive model.

Bisgin et al. demonstrated the use of machine learning in screening for food-contaminating beetles, which currently requires manual microscopic examination. They developed a convolutional neural network (CNN) model trained on high-quality elytral (hardened forewing) images to predict 27 different species of pantry beetles. The model achieved an average accuracy of ~90%. However, several species fell below that average accuracy due to significant intraspecies variation of elytral pattern. This represents an improvement over previous models which will eventually lead to their goal of automated species identification of food-contaminating beetles.

## Genomics

A major challenge in metagenomics is the identification and classification of bacteria in microbial communities that may consist of thousands microbial species. To address this Research Topic, Pham et al. developed a computationally efficient method by using compressed and low-sized genomic signatures of the bacteria to be classified. A modified Bloom filter is used to store k-mers with hash values corresponding to each bacterial species. They showed that most bacteria in many microbiomes can be represented uniquely using the proposed genomic signatures.

As the amount of genome sequencing data increases in the public databases, scalable methods are needed for efficient variant annotation and classification tasks. Li et al. described an updated version of SNPAAMapper, a variant annotation pipeline, with much improved computational efficiency on most updated information. This new version of the SNPAAMapper not only runs faster and more efficiently, it can also classify variants by type of genomic regions (Coding Sequence, Untranslated Regions, upstream, downstream, and intron), predict types of amino acid changes (missense, nonsense, etc.), and prioritize mutation effects (e.g., non-synonymous, synonymous).

Genotype imputation is an important aspect of genome-wide association studies (GWAS). Although deep learning (DL)-based methods have already been developed for this task, it is still challenging to optimize the learning process in DL-based methods in order to achieve high imputation accuracy. Song et al. developed a convolutional autoencoder (AE) model for genotype imputation. Additionally, they implemented a customized training loop by modifying the training process with a single batch loss rather than the average loss over batches. This modified AE-based imputation method was carefully evaluated using multiple real datasets. They found that the modified AE imputation method achieved comparable or better performance than the existing DL-based methods.

Gene prioritization based on molecular function is an important step in utilizing—omics data for understanding human diseases. Nguyen et al. presented a new tool called WINNER for characterizing and prioritizing biomolecules. The tool takes molecular interaction data and expands the network while ranking all nodes by their relevance to other network nodes. These networks can be used to evaluate candidate genes for diseases or proteins from high throughput experiments. The utility of WINNER was evaluated on several diseases such as Alzheimer's disease, breast cancer, myocardial infarctions, and Triple negative breast cancer.

## Protein modeling

Protein structure-function analysis is important for understanding ligand binding properties of proteins as well as for developing new drugs. However, the crystal structures of many proteins are not available in public databases. In one such case, Gokulan et al. modeled VirD4 ATPase, a component of the bacterial type IV secretory system using a variety of bioinformatics and computational tools. The authors hypothesized that the unique insertion regions found in the VirD4 protein could play a role in the flexible movement of the hexameric unit during the relaxosome processing or transfer of the substrate.

## Natural language processing

Machine learning approaches to utilize the vast amount of unstructured text have made tremendous progress in recent years. For example, a graph embedding-based method (MedGraph) was developed by Ebeid to provide a semantic relevance retrieval ranking for biomedical literature indexed in PubMed. Using objective metrics, this a proof-of-concept study provides evidence that graph modeling provides better search relevance than traditional methods.

A fundamental challenge in any social, behavioral or biological study is determination of causality. Further, assessing causality from unstructured text is manual and time-consuming. In their paper, Wang et al. describe a general causal fame work named DeepCausality, which incorporates AI-powered language models, named entity recognition and Judea Pearl's Do-calculus to fulfill different domain-specific applications. They evaluated their method using the LiverTox database to estimate drug-induced liver toxicity (DILI) and validating their results against the American College of Gastroenterology clinical guidelines.

Overall, the papers selected for this Research Topic represent the breadth of computational methods and applications in biomedical and regulatory sciences at the annual MCBIOS conference.

## Author contributions

RH, PM, AT, and ZQ drafted the manuscript. All authors contributed to the article and approved the submitted version.

